# Renal transplantation - substitution therapy in advanced stage uremia

**Published:** 2008-04-15

**Authors:** Sinescu Professor I., Manu M.A., Hârza M., Şerbănescu B., Ştefan B., Cerempei V., Tacu Dorina, Kerezsy Eminee, Bucşa Cristina, Domnişor Liliana, Daia Denise, Constantinescu Ileana

**Affiliations:** * Urological Surgery, Dialysis and Renal Transplantation Center, “Fundeni” Clinical Institute, Bucharest, Romania

**Keywords:** renal failure, donor, renal transplant, immunosuppression

## Abstract

Advanced stage chronic renal failure (CRF) – uremia – represents one of the most severe metabolic “catastrophes” of the organism.

The current therapeutic possibilities consist in: hemodialysis, peritoneal dialysis and renal transplantation.

This paper presents the experience of the Urological Surgery, Dialysis and Renal Transplantation Center of the „Fundeni” Clinical Institute in renal transplantation, the single complete morphological and functional therapeutic option in CRF.

During the last 10 years, renal transplantations with kidneys from brain dead donors (multiorgan harvesting) to an adult (1997), a child (1999), a diabetic recipient (1998) and an anephric child due to bilateral Wilms’ tumor (2005) were performed at „Fundeni” Renal Transplantation Center as a national première.

The immunosuppression protocols are complex, modern and adapted to the immunological risk.

A number of 870 renal transplantations with 82% functionality rate of the graft at 10 years were performed and reported. Among these, 152 transplants were performed using kidneys harvested from brain dead donors.

Owing to obtained results (60% of all transplanted and functional organs in Romania on December 2007) and to its achieved performances, the Fundeni Center represents a reference point on the European map of renal transplant.

The kidney, appreciated by Morgagny as „*viscer elegantissimus*”, is one of the most complex organs, by its morphology and especially, by its function. Thus, the kidney assures the main purifying function of the organism, is decisively involved in the maintenance of both the hydro-ionic equilibrium and the acid-basic balance, accomplishing at the same time the role of a particularly complex and efficient endocrine gland.

Substitution therapy of renal functions includes, essentially, three procedures:

- hemodialysis

- peritoneal dialysis

- renal transplantation.

However, hemodialysis and peritoneal dialysis cannot exceed 12-13% of renal epuration efficacy and consequently, under conditions of long-term utilization of these saving methods, the patient remains nevertheless, both clinically and biochemically, an uremic individual.

The renal transplant is the unique therapeutic way which insures complete morphological and functional substitution, able to restore the endocrine and purifying parameters of the organism, which, in severe chronic renal failure, lead to the greatest metabolic “catastrophe” of the organism.

About 1% of the general population is affected by a severe chronic renal failure and over 50,000 renal transplants are performed on a worldwide level, with a graft functionality of approx. 85% after one year.

Starting with the product of ancient imagination – the Chimera – the fantastic body being formed of parts completely unrelated to each other (a lion’s head, a goat’s body and a dragon’s tail) and continuing with the patrons of surgery, the Holy Fathers without silver coins Cosma and Damian, who have transplanted the lower limb of a Maur in a Christian patient, the efforts made for many decades by researchers and clinicians reached a stage in the replacement of irreversibly affected organs or structures of the organism and have become standards of the medical practice, doubtless the present peak in medical biological sciences.

Interest in renal transplantation existed from the beginning of the 20th century, but the first favourable results were obtained during the sixties, with the inset of immunosuppressive medication use for the prevention and treatment of rejection episodes.

In the course of the following decades, the introduction of immunologic tests (HLA typing, cross-matching etc.), as well as the discovery and use of new immunosuppressive drugs, led to the extension of transplantation activity and to the recording of superior results.

In our country, the first renal transplant was performed in Fundeni Hospital - Bucharest on February 13th, 1980 by Professor E. Proca and his surgical team, in cooperation with anaesthesiologist Dr. A. Popescu and his team.

A significant step forward in Romanian urology was made when a sudden change occurred in the approach and the prospect of the patient with chronic renal failure.

Under financial conditions of the political system of the respective period, this intervention, although representing “a dissidence” was followed by other transplants, reaching a number of 45 up to 1997.

The second stage of evolution in renal transplantation at the Fundeni Clinical Institute began in June 1997, when the present generation assumed responsibility of the department.

During June 1977 – March 2008, 870 renal transplantations were performed at the Fundeni Urologi Surgery, Dialysis and Renal Transplantation Center, offering the leader position to our department in this field.

The achievement of a renal transplant depends to a great extent on the transplanted graft and on the manner in which it is harvested and acquired.

Taking into account that the kidney is a paired organ, functionally oversized, transplantation may be performed not only from cadaver donors, but also from living donors.

The classical axiom “*There is no transplantation without organ donation*” is increasingly present and more imperative from one day to another.

There are three categories of donors:

a) living, genetically related;

b) living, emotionally related (husband-wife, friends, altruistic donors etc.).

c) cadaver donors with two subgroups:

- heart beating, the large majority

- non heart beating.

At present, in Romania, for most renal transplantations, over 80% of the total number of kidneys come from living donors.

Without any doubt, this is due to nothing else than the difficulties which the national network has to overcome in establishing more frequently the diagnosis of brain death, in maintaining the donor under optimum conditions, in obtaining family consent and finally, in harvesting several organs – including kidneys– from this category of donors.

Renal transplantation represents the treatment of choice for patients with end stage chronic renal failure who have no major contraindications.

The *American Society of Transplant Physicians* considers the following situations as absolute contraindications for transplantation:

1) life expectancy under 1 year;

2) recent or untreatable neoplasms;

3) acute or chronic untreatable infections;

4) HIV infections or AIDS;

5) psychosocial problems: uncontrolled major mental disorders, toxic mania, non-compliance etc.;

6) mismatch in the ABO system;

7) positive cross-match between the donor’s lymphocytes and the recipients’ serum.

One of the most important problems in renal transplantation is the assessment, as accurately as possible, of donor and recipient.

The more clinical, anatomical and immunological parameters are adapted, so as to offer a closer match, the more favourable will be the results of this miraculous therapy, aimed at controlling redoubtable diseases.

It is useless to mention that the quality of results and the post transplantation course depend to a great extent on the precision and accuracy of the operative act, on the biology of the organism in which the graft should function and, last but not least, on the equilibrium at any time fragile of the immunosuppressive therapy.

Unfortunately, the number of potential recipients exceeds significantly that of donors, so during the last years stress was more and more laid on the notions of marginal donor and recipient for the renal transplantation.

The ideal kidney donor for renal graft should meet the following criteria:

* Immunological criteria: blood group, HLA typing, negative cross-match; 

* Non-immunological criteria:

- voluntary, mentally normal donor, 1st degree related to the recipient, aged between 18 and 65 years;

- anatomy of kidney vessels and urinary tract within normal limits;

- normal anatomical disposition of renal arteries and veins;

- donor’s nephrectomy should not affect his state of health;

- donor should not be a carrier of infectious agents: hepatitis B or C virus, cytomegalovirus or HIV.

By considering these rules as ideal selection criteria of a renal donor, it is obvious that the number of those who can satisfy these conditions is low.

In order to widen the indication of potential donor, the concept of “relative and absolute contraindications” of kidney donation were taken into account in renal transplantation. It is not the case to discuss absolute contraindications but the relative contraindications superimpose themselves very well on the concept of marginal donor. An important criterion which should be taken into account refers to the anatomy of the kidney and of the urinary tract. The ideal situation is represented in the atlas of anatomy, but anatomical variants are multiple and are not a contraindication for transplantation. The greatest anatomical diversity is established by the renal vessels. The evaluation of all donors in our Center was complete and standard protocols were respected. We have never omitted, in living donors, to evaluate the renal pedicle for a logical and correct selection of the kidney which will be taken for transplant.

Thus, all the donors from our Center were examined by ultrasonography and Doppler scanning for renal pedicle, and the vessels were evaluated by arteriography (global aortography, followed by selective renal arteriography), spiral CT scan or angio-MRI. By means of all these preoperative investigations are visualized the anatomy of the renal pedicle, the main renal artery, the anatomical variants, the codominant arteries, the early branches, the aberrant and accessory arteries and the multiple renal veins, elements which range the donor in the marginal category.

Using these investigations, we have the possibility to adequately prepare the recipient and his vessels, we shorten the warm and cold ischemia times and we are always able to harvest the kidney which is from all the viewpoints easier to graft with maximum security for the donor.

The harvesting act from a living donor, without preoperative investigation of the renal pedicle, represents a hazardous and a non-medical attitude.

In the included figures some anatomical variants of the renal vascularisation are presented, which lead to the concept of marginal donor, as well as the complex method by which the renal pedicle is preoperatively explored in our Center by angiography, 3D CT scan, both compared with the intraoperative vascular anatomy.

In our experience, the selective renal angiography remains for the time being, although invasive, the method which implies the minimum possibilities of a diagnosis error.

We have always refused to harvest kidneys from living donors with more than two arteries, not because the current techniques do not allow vascular reconstruction, but because the more laborious is the mentioned procedure, the higher is the risk of potential postoperative complications.

**Figure:**
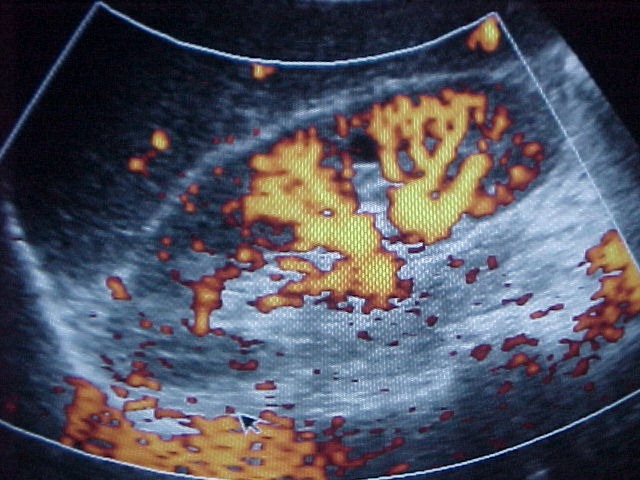
Power Echo Doppler (the aspect of the intrarenal arterial vascularisation).

**Figure:**
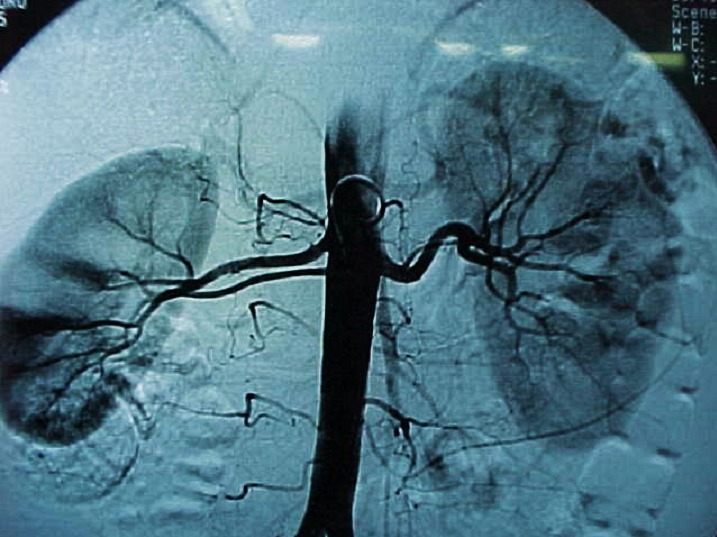
Standard renal arteriography.

**Figure:**
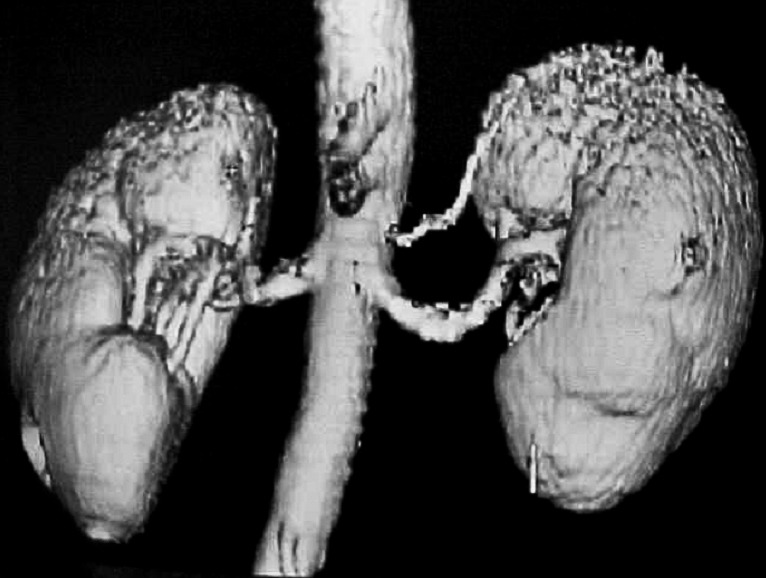
Left superior accessory vessel (3D spiral angio-CT scan).

**Figure:**
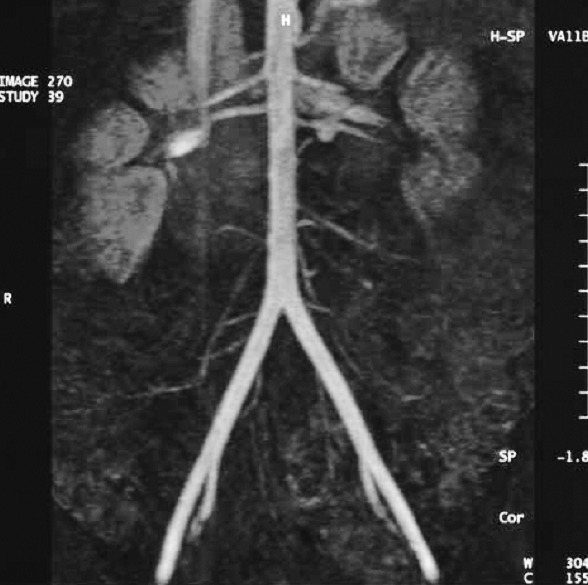
Angio-MRI.

The moral and medical responsibility is immense in the moment of decision to harvest a kidney from a living donor and can be justified only by a very good result in the recipient; anyway, a technical cause of the graft dysfunction can never be accepted. It is obvious that the minimal lombotomy for renal harvesting for transplantation represents the classical and optimal indication because it is easy to perform, cheap and does not require a particular specialization.

In 718 nephrectomies performed on living donors, major complications were not recorded, the morbidity of donors being in accordance to international parameters, the mortality was 0 and the renal function of the donors remained normal and stable up to now.

In regard to the harvest from brain dead donors, we mention some reference data, which inscribe themselves on the map of organs and tissue harvesting in Romania: June 22nd, 1997 – the first multiorgan harvesting (kidney and liver) from a brain dead donor, with the performance of renal transplantations in adults, with favourable course, and on January 3rd, 1998 – promulgation of the “Law regarding organs and tissues harvesting” which offered a new approach for renal transplantation from brain dead donor in Romania.

For the last 10 years, 152 transplants with kidneys from brain dead donors were performed, of which 144 to adults and 8 to children.

The assessment of the brain dead donor mandatorily includes laboratory tests: blood group and Rh, HLA typing, serum and urinary biological check-up and screening for viral and bacterial infections. A check-up of the main organs is required and the donors’ selection is done according to the “100 minimum criteria”: systolic blood pressure >100 mmHg, pO2>100 mmHg and hourly urinary output >100 ml.

We present some recommendations regarding the harvesting of organs from brain dead donors according to the European Association of Urology Guidelines: during the multiorgan harvesting good coordination and cooperation between the different surgical teams are essential. After thoracic organs and liver removal, if there is no consensus regarding the removal of pancreas it is advisable to take in block both the kidney and the pancreas and to separate them on the dissection table; in the case of multiorgan harvesting, the kidneys are the last organs which are harvested; the correct placing of the aortic cannula is essential for the *in situ* perfusion; the multiple intra-abdominal organs harvesting by a total *en bloc* abdominal evisceration technique prevents warm ischemia and injury by traction of the vascular tree. A good preservation and an optimal immunosuppression are interdependent of each other; a good preservation will insure normal and immediate renal function of the graft.

Renal transplantation from a living donor offers ideal conditions for organ preservation. Results in transplantation from living donors are better than those from brain dead donors, which nonetheless still represent the majority of renal graft sources for transplantation.

In using organs from brain dead donors, the improvement of clinical care of donors before and during organ harvesting, optimal preservation and storage conditions and optimal immunosuppression contribute to a better efficiency of the renal transplantation.

During living donor nephrectomy, the extracellular fluid volumes are maintained through parenteral supply. Manitol is frequently administered for the maintenance of an adequate diuresis. Vascular injuries are avoided during kidney mobilisation. Cooling off the kidney begins after only a few seconds of warm ischemia. Total ischemia usually lasts 15-30 minutes and its duration should preferably not exceed one hour, if the operations on the donor and the recipient are simultaneously carried out.

An excellent preservation of the renal functions is assured by a simple cooling in ice, associated with an intra-arterial irrigation with a cold preservation solution.

In the case of a brain dead donor, kidneys are first washed out of blood with a cold solution, often beginning *in situ* in the donors with cardiac activity. Afterwards, the kidneys are immersed in a cold solution. Cold washing removes the blood and augments the cooling rate.

The cooling by *in situ* washing may lower the organ’s temperature to less than 10°C within 3-5 minutes, in comparison with 20 minutes in the case of surface cooling.

The recommended procedure for kidneys harvested from cadaver donors consists in immersing the organ into saline and afterwards in its washing with a preservation solution.

The kidney is then surrounded by a perfused solution and placed into a sterile container (usually double plastic bags) and cold stored in an isolating container.

Perfusion and preservation solutions for renal grafts which can assure a cold ischemia for approximately 24-36 hours without causing graft failure are Collins/Euro-Collins, Bretschneider’s HTK, Wisconsin University, Soltran, the Celsior solution etc. By observance of these protocols, the courses of the transplanted patients with kidneys from brain dead donors are ranged within international parameters. The whole harvesting activity, preservation, transport and transplantation activity of the renal grafts from brain dead donors was performed by surgeons of our Center who were specialized abroad.

From cadaver donors, when kidneys are harvested *en bloc*, a significant dissection is required, the uretheral arterial source being compulsorily maintained.

The kidney harvested from the living donor may have several additional arteries; in this case, reconstruction is performed *in vivo*, the arterial stoma forming a single trunk, a termino-lateral or a termino-terminal anastomosis.

It is imperative to assure a revascularization of the inferior polar artery, because it very often represents the single uretheral arterial source. In the existence of more than a single renal vein, the small veins will be ligated.

If two renal veins have the same calibre, one of them will not be ligated because of the risk of venous infarct and it is preferable to achieve a common trunk before performing the anastomosis.

A short renal vein may be lengthened by means of a patch of inferior vena cava.

The renal transplantation is a major surgical procedure, which implies a vascular and a urological surgical skill.

The recipient is a high-risk patient with associated morbidities (diabetes, cardiovascular disease etc.). The recipient will exhibit a significant degree of platelet dysfunction and an increased susceptibility to bleeding. The recipient’s acceptance for renal transplantation should be submitted to a careful analysis and to a detailed clinical examination, in order to assure ourselves that there is no immediate contraindication for major surgical procedure, like hydro-electrolytic imbalance and increased potassium level. The patient may need a dialysis session preceding the surgical procedure.

The immunosuppression, regardless of protocol, is usually initiated before the operation.

Usually, the left kidney will be placed into the right iliac fossa and the right kidney into the left iliac fossa, in order to anteriorly put the renal pelvis and ureter, this way facilitating the reconstruction of the urinary tract. If the patient has undergone a peritoneal dialysis, the transplanted kidney will be placed in the opposite side of the dialysis catheter. In the case of polycystic kidneys, the smaller kidney side will be chosen. In children, in whom vascular anastomoses are performed with the aorta and the vena cava, it is preferable to use the right side since the kidney is placed behind the caecum and the ascending colon. Depending on the kidney’s position it will be established which arterial or venous anastomosis will be first performed. Generally, if the renal artery will be anastomosed to the internal iliac artery, the arterial anastomosis will be done first.

If the renal artery will be anastomosed termino-laterally to the external iliac artery, it is preferable to follow the venous anastomosis. The renal vein may be anastomosed laterally or medially to the external iliac artery, usually termino-laterally with the external iliac vein.

After renal revascularization, the kidney is placed in the final position and implantation in the urinary bladder will be performed.

The operative procedures described above are illustrated by the following pictures:

**Figure:**
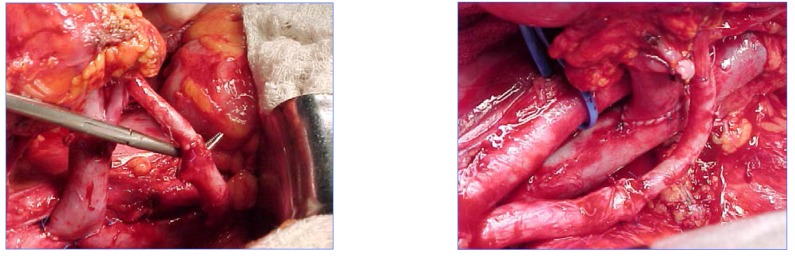
Renal artery – hypogastric artery termino-terminal (T-T) anastomosis and renal vein – external iliac vein termino-terminal (T-T) anastomosis.

We increased the number of transplants with 31% using special anastomotic techniques.

Preoperative vascular evaluation is compulsory.

In the case of equal kidneys, the technically easiest one will be harvested. In the case of unequal kidneys the weak one will be chosen.

The smaller arterial sources, which supply less than 5% of the renal parenchyma, may be sacrificed and it should be known that laborious salvation manoeuvres with an uncertain result, will inadmissibly prolong ischemia times; the damage caused by them exceeds significantly the expected benefit.

The accurate appreciation of the revascularization method is the key of success in transplantation.

We illustrate in the following pictures the results in abnormal vascular kidney transplant:

**Figure:**
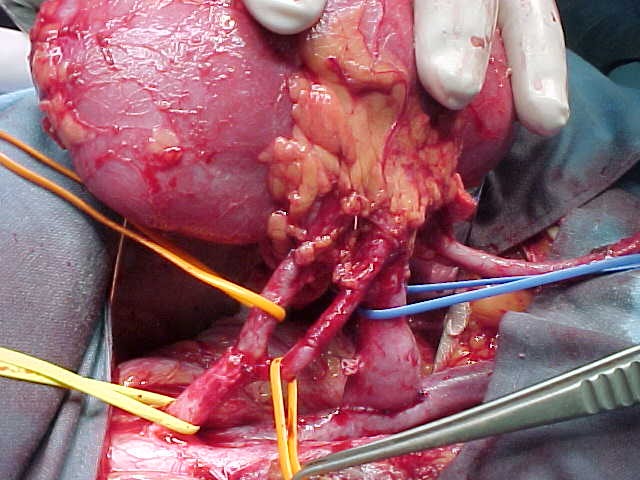
Angioplasty with single trunk of codominant renal arteries.

**Figure:**
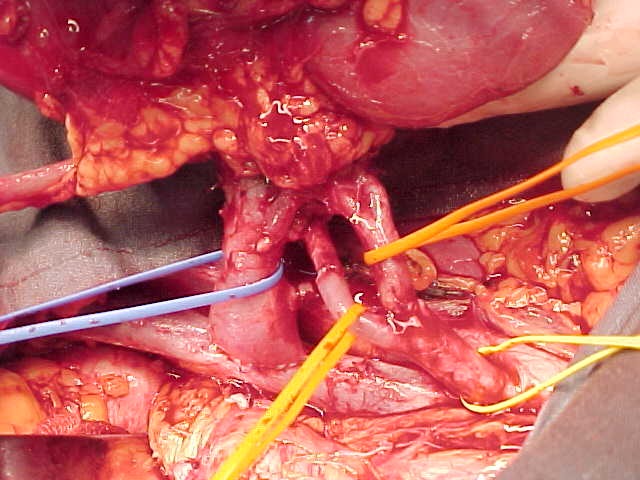
Double anastomosis of the codominant branches to the hypogastric artery.

Immunosuppressive treatment in the organ transplantation has the main role in maintaining the balance between the graft and the immune response.

It was observed that the risk of graft loss due to surgical or immunological reasons or drug toxicity is highest during the first months post transplantation; after the first year, graft survival and its loss rate have a linear evolution.

The improvement of renal transplantation outcomes has been simultaneous with the development of transplant immunology and immunosuppressive therapy. 

First attempts to suppress rejection started in the late 50’s and early 60’s using total body irradiation and steroids, and then 6MP derivates. In the early 70’s, Welcome laboratory synthesizes a precursor of 6MP with good clinical efficacy and less toxicity: Azathioprine. Polyclonal anti-lymphocyte preparation is also introduced in the 70’s and the transplantation field enters the 80’s with a remarkable 70% success rate at one year. Cyclosporine introduction in 1983 marks the beginning of modern immunosuppression and continued improvement in transplant success. It also marks the first attempts to minimize steroids usage and even to withdraw them in an attempt to lower the comorbid load induced by the use of these drugs. Neoral, the micro emulsion of cyclosporine, gives it independence from biliary salts for absorption. Tacrolimus is later introduced as an alternative to cyclosporine, and later, in 1996, the more specific antimetabolite drug MMF is introduced as a better alternative to Azathioprine. Anti-IL2 receptor blockers and Thymoglobulin expand the spectrum of monoclonal and polyclonal antibodies and allow further reduction of acute rejection rates, leading to a greater than 90% graft survival at one year since 2000.

 In spite of the good short and medium term results, immunosuppression is involved in the pathogenesis of chronic graft nephropathy and can generate morbidity and mortality in these patients by itself.

 Because of the more important risk of rejection in the first period after transplantation and the higher prevalence of infection and neoplasia in the late period, immunosuppression has to be changed according to allograft age. 

Taking into account these aspects, the immunosuppressive therapy varies according to the time elapsed since transplantation; three important periods of immunosuppression may be distinguished:

- *the induction period* – during which a medication of a high immunosuppressive capacity is used for a short period of time, its objective being to lower the risk of acute rejection during first months post transplantation;

- *the early maintenance period* (the first months post transplantation) – characterized by the establishment of individual immunosuppressive regimes (with drugs and doses specific to each recipient);

- The period of chronic maintenance – characterized by the administration of relatively constant low doses (minimal effective doses) of immunosuppressive drugs, which will be modified only in the case of complications specific to this period (late acute rejections, infections, neoplasms etc.)

The most important immunosuppressive drugs used in renal transplantation are:

1. Glucocorticoids: they can be used in every moment of immunosuppression: induction, maintenance and acute rejection therapy.

2. Small-molecule Drugs:

- Immunophilin-binding drugs:

a. Calcineurin inhibitors: this class of drugs is widely used in renal transplantation. The main immunosuppressive action is a decrease in IL2 synthesis, followed by inhibition of T cell proliferation and maturation. The most important adverse effects are nephrotoxicity, metabolic disorders (dislipidemia, hyperuricemia, dibetes), arterial hypertension.

- Cyclophilin-binding drugs: Cyclosporine

- FKBP12-binding drugs: Tacrolimus

b. Target-of-rapamycin inhibitors: Sirolimus or Rapamycin is a macrolide antibiotic derived from a Streptomyces fungus. Rapamycin offers a completely different mechanism of action: by binding to the FKBP, it inhibits the proliferative message of the third signal, usually transmitted from the IL2 R. Sirolimus has antiproliferative and antineoplastic properties. Also, Sirolimus can offer an alternative to calcineurin inhibitors in chronic allograft nephropathy patients.

* Inhibitors of nucleotide synthesis:

- Purine Synthesis (IMPDH) inhibitors: MMF, Mycophenolate Sodium - precursors of Mycophenolic acid (Penicillium sp) blocks IMPDH and decreases GMP production. These drugs are selective blockers of T and B cell proliferation. Main side effects are GI toxicity, bone marrow suppression

- Pyrimidine Synthesis (DHODH) inhibitors: Leflunomide, FK778-in development

- Antimetabolites: Azathioprine was replaced by MMF in immunosuppressive protocols.

- Sphingosine-1-phosphate receptor antagonist: FYT720

3. Protein Drugs

- Depleting Antibodies are used as an induction therapy and in severe acute rejection. 

Severe cytopenia and anaphylactic reaction can occur in the course of this therapy. Also, they increase the risk of malignancy and opportunistic infections.

- Polyclonal Antibody: horse or rabbit anti-thymocyte globulin

- Mouse monoclonal anti-CD3 antibody: Muromonab-CD3

- Humanized monoclonal anti-CD52 antibody: Alemtuzumab

- B-cell-depleting monoclonal anti-CD20 antibody: Rituximab. 

* Nondepleting antibodies and fusion proteins are used just in induction therapy

* Humanized or Chimeric monoclonal anti-CD25 antibody: Daclizumab and Basiliximab. These drugs have a very good tolerance and they do not increase the risk of malignancy and opportunistic infections.

* Fusion protein: CTLA-4-Ig (LEA29)- in development.

* Intravenous Immunoglobulins – are used in hyper sensitised patients and in humoral rejection reactions.

Our Center has an important experience in the handling and monitoring of these drugs which is reflected in good short and long time outcomes.

In our Center, for a period of 10 years: 1997- March 2008, 870 renal transplants were performed, with an average of 100 procedures in the last 4 years, with a maximum of 116 in 2007, a fruitful activity similar to western countries’ centers.

There were 718 transplants from living donors (83%) and 152 transplants from brain dead donors (17%), 821 adults and 49 children; 37 recipients were diabetic patients.

During these 10 years we recorded many remarkable national level achievements, among which we mention the first renal transplantation to a diabetic recipient – 1998 (refused in Great Britain and Germany) and the first renal transplantation to a recipient with pelvic neoplasm (surgically treated, with chemotherapy, radiotherapy, dialysis etc.); 1999 – the first transplant to a child from a cadaver donor was performed and in 2000 – the first Laboratory for Transplant Immunology and Molecular Biology was organised.

Likewise, it is noteworthy to remind that, during 1998 – 2002, renal transplant teams of the Centers of Oradea and Iaşi were trained both by specialised courses in our Center and started to perform efficient interventions in Oradea (3 renal transplants in 1998 and 1999) and in Iaşi (8 renal transplants in 2000 and 2001).

The first renal transplantation to an anephric child with bilateral Wilms’ tumor (bilateral nephrectomy, chemoherapy, radiotherapy, dialysis etc.) was performed in our country in 2005, for the first time in Romania.

Renal transplantation represents the typical example of a multidisciplinary activity along the evolution of the uremic patient, from the moment of renal failure diagnosis and up to the morphological and functional stable post transplant result, correlating the activity of at least seven medical specialities (Dialysis – Nephrology, Immunology, Medical Imaging, Urological Surgery, Intensive Care Unit, laboratories of Hematology, Biochemistry, Bacteriology, Pathology and Coordination), each with a special role for the therapeutic success. Their intervention is sequential, their activity is correlated, many of the mentioned specialists being permanently involved also in monitoring the post transplant course.

The Renal Transplantation Department which functions in our Center is the most unique autonomous department in Romania, organized as follows: it has 32 beds, of which 11 intensive care beds, 2 hemodialyzer equipments, 2 transplant anaesthesiologists, 3 nephrologists and specialized nursing stuff. The surgical activity is performed totally by urologists of our Center.

To our center there is attached an Immunology and Molecular Biology Laboratory which achieves the complex and complete immunological assessment for all the cases and elaborates modern immunosuppression protocols, adapted to the degree of immunological risk.

The post transplantation course and the results are expressed in the following percentages: - Graft functionality at 1 year 96%

 - Graft functionality at 5 years 84%

- Global graft functionality al 10 years (1997-2007) 82%.

For comparison, we present *The U.S. Organ Procurement and Transplantation Network and The Scientific Registry of Transplant Recipients* data regarding graft survival at 3 months, 1 year, 3 years, 5 years and 10 years post transplant (2006 report):

**Table:** 

			Survival (%)		
*Kidney*:	3 months	1 year	3 years	5 years	10 years
Cadaver donor	94,3%	89,5%	78,6%	67,1%	40,8%
Living donor	97,2%	95,1%	88,4%	80,3%	56,5%

Within national context, in December 2007 there were 1,180 recipients under immunosuppressive therapy post organ transplantation (kidney, liver, heart) in Romania, 702 of these recipients were renal transplanted in our Center, respectively 60% of functional transplanted organs in Romania that belong to the Fundeni Urological Surgery, Dialysis and Renal Transplantation Center.

A brief analysis of our centre’s experience allows us to assert that it is the most experimented, the greatest and the most efficient in Romania, a reference centre on the European map of renal transplantation in terms of organization, competence, performance and results.
